# Understanding integrase inhibitor resistance in Angola: a comparative analysis of INSTI-naive and INSTI-experienced individuals

**DOI:** 10.1093/jac/dkag355

**Published:** 2026-11-03

**Authors:** Bárbara Pocongo, Maria Furtado, Cláudia Bernardi, Lucrécia Jacinto, Aulória José, Jaime Urica, Rosa Torres, Francisco Vunge, Beatriz Almeida, Michael R Jordan, Carlos Jesus, Monick Lindenmeyer Guimarães, Cíntia Mayumi Ahagon, Giselle Lopes, Luís Brígido, Seth Inzaule, Ana B Abecasis, Ricardo Parreira, João Piedade, Victor Pimentel

**Affiliations:** Instituto Nacional de Luta Contra SIDA (INLS), Ministério da Saúde, Luanda, Angola; Global Health and Tropical Medicine, GHTM, LA-REAL, Instituto de Higiene e Medicina Tropical, IHMT, Universidade NOVA de Lisboa, Lisboa, Portugal; Instituto Nacional de Luta Contra SIDA (INLS), Ministério da Saúde, Luanda, Angola; Instituto Nacional de Luta Contra SIDA (INLS), Ministério da Saúde, Luanda, Angola; Instituto Nacional de Luta Contra SIDA (INLS), Ministério da Saúde, Luanda, Angola; Instituto Nacional de Luta Contra SIDA (INLS), Ministério da Saúde, Luanda, Angola; Instituto Nacional de Luta Contra SIDA (INLS), Ministério da Saúde, Luanda, Angola; Instituto Nacional de Luta Contra SIDA (INLS), Ministério da Saúde, Luanda, Angola; Instituto Nacional de Luta Contra SIDA (INLS), Ministério da Saúde, Luanda, Angola; Global Health and Tropical Medicine, GHTM, LA-REAL, Instituto de Higiene e Medicina Tropical, IHMT, Universidade NOVA de Lisboa, Lisboa, Portugal; Global Health and Tropical Medicine, GHTM, LA-REAL, Instituto de Higiene e Medicina Tropical, IHMT, Universidade NOVA de Lisboa, Lisboa, Portugal; Tufts University School of Medicine, Boston, MA, USA; Laboratório de AIDS e Imunologia Molecular, Instituto Oswaldo Cruz, Rio de Janeiro 21045-900, Brazil; Laboratório de AIDS e Imunologia Molecular, Instituto Oswaldo Cruz, Rio de Janeiro 21045-900, Brazil; Instituto Adolfo Lutz, Centro de Virologia, São Paulo, SP, Brazil; Instituto Adolfo Lutz, Centro de Virologia, São Paulo, SP, Brazil; Instituto Adolfo Lutz, Centro de Virologia, São Paulo, SP, Brazil; Amsterdam Institute for Global Health and Development, and Department of Global Health, Amsterdam UMC, University of Amsterdam, Amsterdam, The Netherlands; Global Health and Tropical Medicine, GHTM, LA-REAL, Instituto de Higiene e Medicina Tropical, IHMT, Universidade NOVA de Lisboa, Lisboa, Portugal; Global Health and Tropical Medicine, GHTM, LA-REAL, Instituto de Higiene e Medicina Tropical, IHMT, Universidade NOVA de Lisboa, Lisboa, Portugal; Global Health and Tropical Medicine, GHTM, LA-REAL, Instituto de Higiene e Medicina Tropical, IHMT, Universidade NOVA de Lisboa, Lisboa, Portugal; Global Health and Tropical Medicine, GHTM, LA-REAL, Instituto de Higiene e Medicina Tropical, IHMT, Universidade NOVA de Lisboa, Lisboa, Portugal

## Abstract

**Background:**

Africa remains a major epicentre of the global HIV epidemic, with Central Africa exhibiting extensive HIV-1 genetic diversity that may influence the emergence and transmission of drug resistance mutations. Following World Health Organization recommendations, Angola initiated the national transition to dolutegravir (DTG)-based antiretroviral therapy (ART) in 2021. This study evaluated the frequency, patterns and temporal trends of HIV-1 drug resistance mutations before and after DTG implementation (2019–23), while also characterizing HIV-1 genetic diversity across Angolan provinces outside Luanda.

**Methods:**

A retrospective observational analytical study comparing pre- and post-Integrase strand transfer inhibitor (INSTI) introduction periods was conducted using plasma samples and dried blood spot cards from 17 provinces of Angola.

**Results:**

Of the 201 samples analysed, 102 were successfully sequenced for at least one coding region [protease (*n* = 65); reverse transcriptase (*n* = 66); integrase (IN) (*n* = 86)], comprising 48 INSTI-naive people living with HIV and 54 INSTI-experienced people living with HIV samples. Subtype C was the most prevalent (21.6%), followed by unique recombinant forms (20.6%), A1 (15.7%), F1 (13.7%), CRF02_AG (10.8%), G (8.8%) and other subtypes or recombinants (8.8%). Resistance to non-nucleoside reverse transcriptase inhibitors was the most common (68.2%; 95% CI 56.2–78.2), followed by nucleoside reverse transcriptase inhibitors (48.5%; 95% CI 36.8–60.3). Resistance to protease inhibitors (PIs) and INSTIs was lower, observed in 1.5% (95% CI 0.3–8.2) and 3.5% (95% CI 1.2–9.8), respectively.

**Conclusions:**

In this analysis, substantial HIV-1 genetic and epidemiological complexity was observed. In the context of the rapid scale-up of DTG-based ART, continued surveillance of INSTI resistance mutations has become increasingly important to inform evidence-based public health strategies aimed at preventing the transmission of drug-resistant HIV strains and optimizing ART regimens at the population level.

## Introduction

Advances in antiretroviral therapy (ART) have enabled people living with HIV (PLHIV) to enjoy longer life expectancy and an improved quality of life. Despite these advances, the ongoing development of more robust and durable drug regimens remains critical to avoid the long-term risk of drug resistance and treatment failure.^1^

Integrase strand transfer inhibitors (INSTIs) are a class of antiretroviral drugs that inhibit the viral cDNA strand transfer reaction catalysed by HIV INT, thereby preventing retroviral integration into the host genome. The introduction of INSTIs into ART regimens has transformed the clinical management of HIV-1 infection. Among these agents, dolutegravir (DTG) stands out for its potent antiviral activity, high genetic barrier to resistance and favourable safety profile.^2^ In 2018, the World Health Organization (WHO) recommended a DTG-based regimen as the preferred first-line therapy in low- and middle-income countries, particularly in sub-Saharan Africa, where high levels of resistance to non-nucleoside reverse transcriptase inhibitors (NNRTIs) had compromised treatment efficacy.^2^ However, HIV drug resistance (HIVDR) remains an emerging threat to the long-term success of HIV treatment programmes and epidemic control.^3,4^

Africa remains a key epicentre of the global HIV epidemic, and Central Africa harbours remarkable genetic diversity of viral subtypes.^5^ This diversity may influence the emergence and patterns of resistance mutations, emphasizing the need for region-specific molecular surveillance.^2,6^

Angola implemented free ART in 2004 as part of its national HIV response strategy, achieving an estimated ART coverage of 48%, corresponding to approximately 180 000 PLHIV.^7^ Despite progress, this level of coverage remains below the regional average for sub-Saharan Africa, reflecting persistent challenges in healthcare access and ART distribution.^8,9^ ART coverage in Angola remains suboptimal due to regional disparities, economic instability and interruptions in drug supply, while low adherence to ART further contributes to virological failure and may accelerate the emergence of HIV drug-resistant variants.^10–12^ Following WHO recommendations, Angola initiated the national transition to DTG-based ART in 2021.^13^

This study aims to evaluate the frequency, patterns and temporal trends of HIV-1 drug resistance mutations (DRMs) among adult and paediatric patients receiving ART in Angola before and after the national introduction of DTG-based regimens (2019–23) and to characterize the genetic diversity of HIV-1 across provinces outside Luanda, where surveillance of INT inhibitor resistance remains scarce.

## Materials and methods

### Study design and setting

A retrospective observational analytical study comparing pre- and post-INSTI introduction periods was conducted using plasma samples and dried blood spot (DBS) cards from 17 provinces of Angola. The selected samples, collected between 2019 and 2023, were stored at the Molecular Biology Reference Laboratory of the National Institute for the Fight Against AIDS in Luanda. A convenience sample of 201 specimens (134 DBS and 67 plasma) used for routine viral load tests from individuals receiving ART with viral non-suppression (viral load >1000 copies/mL) was analysed. The samples were categorized into two groups according to INSTI treatment exposure: 92 samples for INSTI-naive PLHIV (INP) and 109 samples for INSTI-experienced PLHIV (IEP). Individuals younger than 15 years old were considered children, whereas those aged 15 years and older were considered adults.

### Extraction, PCR amplification and sequencing of HIV-1 RNA and DNA

Viral RNA was extracted from plasma and DBS samples using the QIAamp^®^ Viral RNA Mini Kit (QIAGEN, Hilden, Germany) according to the manufacturer’s instructions. Reverse transcription of the extracted RNA was performed following a previously described protocol.^14,15^

Two rounds of nested PCR were performed to amplify the HIV-1 *pol* gene regions comprehending protease/reverse transcriptase (PR/RT; HXB2 positions 2253–3353) and INT (HXB2 positions 4239–5096) coding sequences. The resulting nested-PCR products were analysed by 1% (w/v) agarose gel electrophoresis, purified using the Wizard^®^ SV Gel and PCR Clean-Up System (Promega, Madison, WI, USA) and subsequently sequenced using the Sanger sequencing method with specific primers, as previously described.^14,15^ Sequencing was performed using an ABI 3730xl automated sequencer with the BigDye^®^ Terminator v3.1 Cycle Sequencing Kit (Applied Biosystems, Madison, WI, USA).

### Analysis of HIV-1 genetic diversity and profile of resistance mutations

The generated FASTA sequence files were processed using RECALL v2.5 software^16^ for sequence assembly and editing. HIV-1 subtype classification was performed using the COMET subtyping tool.^17^ DRMs were identified using the Stanford University HIV Drug Resistance Database (HIVdb) v10.1 (https://hivdb.stanford.edu/).

### Statistical analysis

Statistical analyses were performed using IBM SPSS Statistics v29 (IBM Corp., USA). Quantitative variables were summarized using descriptive statistics, including sample size (*n*) and median with interquartile range (IQR; 25th–75th percentiles). Categorical variables were presented as frequencies and proportions and compared using the Fisher’s exact test. Statistical significance was defined as a *P* value <0.05 for all analyses.

### Ethical approval

This study was conducted in accordance with ethical principles for medical research involving human subjects and received formal approval from the Ethics Committee of the Angolan Ministry of Health No. 039/CEM/2023. All procedures involving human samples were performed following the guidelines and regulations established by the Committee to ensure the protection of participants’ rights, confidentiality and safety.

## Results

Out of the 201 participants enrolled, 42 (20.9%) were children and 159 (79.1%) were adults. The median age was 34.0 years (IQR 18–42.5) and 76.6% were female; 92 (45.8%) samples were from INPs, while the remainder 109 (54.2%) were from IEPs; 59.7% of the samples were from the northern region of Angola (Luanda, Malanje, Cuanza Norte, Cabinda, Uíge and Zaire provinces), 21.4% from the central-southern region (Bié, Huambo, Cuanza Sul, Benguela, Cunene, Huíla, Namibe and Cuando-Cubango provinces) and 18.9% from the eastern region (Lunda Norte, Lunda Sul and Moxico provinces). The average HIV-1 RNA load in the samples analysed was 5.50 log_10_ copies/mL (Table 1).

**Table 1.** Socio-demographic characteristics of the population

Independent variable	*n* (%)	Treatment status			Independent variable	*n* (%)	Treatment status		
		INP (%)	IEP (%)	*P* value			INP (%)	IEP (%)	*P* value
Overall sequenced samples	201 (100)	92 (45.8)	109 (54.2)		Amplified samples	102 (100)	48 (47.1)	54 (52.9)	
Age, years, median (IQR)	34 (18–42.5)	33 (15–40.7)	36 (19.5–43.5)		Age, years, median (IQR)	38 (18–42.5)	38 (15–40.7)	38 (19.5–43.5)	
<15 years	42 (20.9)	23 (54.8)	19 (45.2)	0.189	<15 years	21 (20.6)	12 (57.1)	9 (42.9)	0.152
>15 years	159 (79.1)	69 (43.4)	90 (56.6)		>15 years	81 (79.4)	36 (44.4)	45 (55.6)	
Male	47 (23.4)	21 (44.7)	26 (55.3)	0.864	Male	28 (27.5)	15 (53.6)	13 (46.4)	0.467
Female	154 (76.6)	71 (46.1)	83 (53.9)		Female	74 (72.5)	33 (44.6)	41 (55.4)	
VL, media, Log_10_	201 (5.50)	92 (5.43)	109 (5.46)		VL, media, Log_10_	102 (5.65)	48 (5.65)	54 (5.58)	
VL <5 Log_10_	45 (22.4)	22 (48.9)	23 (51.1)	0.882	VL <5 Log_10_	11 (10.8)	9 (81.8)	2 (18.2)	<0.0001
VL >5 Log_10_	156 (77.6)	69 (44.2)	87 (55.8)		VL >5 Log_10_	91 (89.2)	39 (42.9)	52 (57.1)	
Northern region	120 (59.7)	52 (43.3)	68 (56.7)	0.095	Northern region	70 (68.6)	28 (40)	42 (60)	0.043
Central southern region	43 (21.4)	22 (51.1)	21 (48.8)		Central southern region	23 (22.5)	15 (65.2)	8 (34.8)	
Eastern region	38 (18.9)	18 (47.3)	20 (52.6)		Eastern region	9 (8.82)	5 (55.6)	4 (44.4)	

IEP, INSTI-experienced PLHIV; INP, INSTI-naive PLHIV; IQR, (25–75 percentiles); VL, viral load.

Of the 201 samples analysed, 102 (54 plasma and 48 DBS) were successfully sequenced for at least one coding region [PR (*n* = 65); RT (*n* = 66); IN (*n* = 86)], and 50 samples amplified across the three regions, comprising 48 INP and 54 IEP samples (Table 1). Overall, Subtype C was the most prevalent subtype (21.6%), followed by unique recombinant forms (URFs; 20.6%), A1 (15.7%), F1 (13.7%), CRF02_AG (10.8%), G (8.8%) and other subtypes or recombinants (8.8%). Resistance to NNRTIs was most common (68.2%; 95% CI 56.2–78.2), followed by NRTIs (48.5%; 95% CI 36.8–60.3). Resistance to PIs and INSTIs was lower, observed in 1.5% (95% CI 0.3–8.2) and 3.5% (95% CI 1.2–9.8), respectively (Figure 1a and b). The prevalence of resistance-associated mutations differed between groups, with higher frequencies observed in INP compared to IEP, for NNRTIs (76.3% versus 57.1%; *P* = 0.09) and NRTIs (65.7% versus 25.0%; *P* = 0.001), while INSTI resistance was low in both groups (5.3% versus 3.6%; *P* = 0.74). PI resistance was observed only in IEP (3.6%). Analysing the two groups, the most prevalent NNRTI mutations were K103N/S/T (40.9%), G190A/E (19.7%) and E138A/G (12.1%), while NRTI mutations were M184V (37.9%), K70R/E/Q (7.6%) and T215Y/F/S/V (6.1%). INSTI major mutations were rare (G140R, Q148H and R263K; 1.2% each; Figure 1c), and PI mutations included I54V and V82A (1.5% each; Figure 1d).

**Figure 1.**
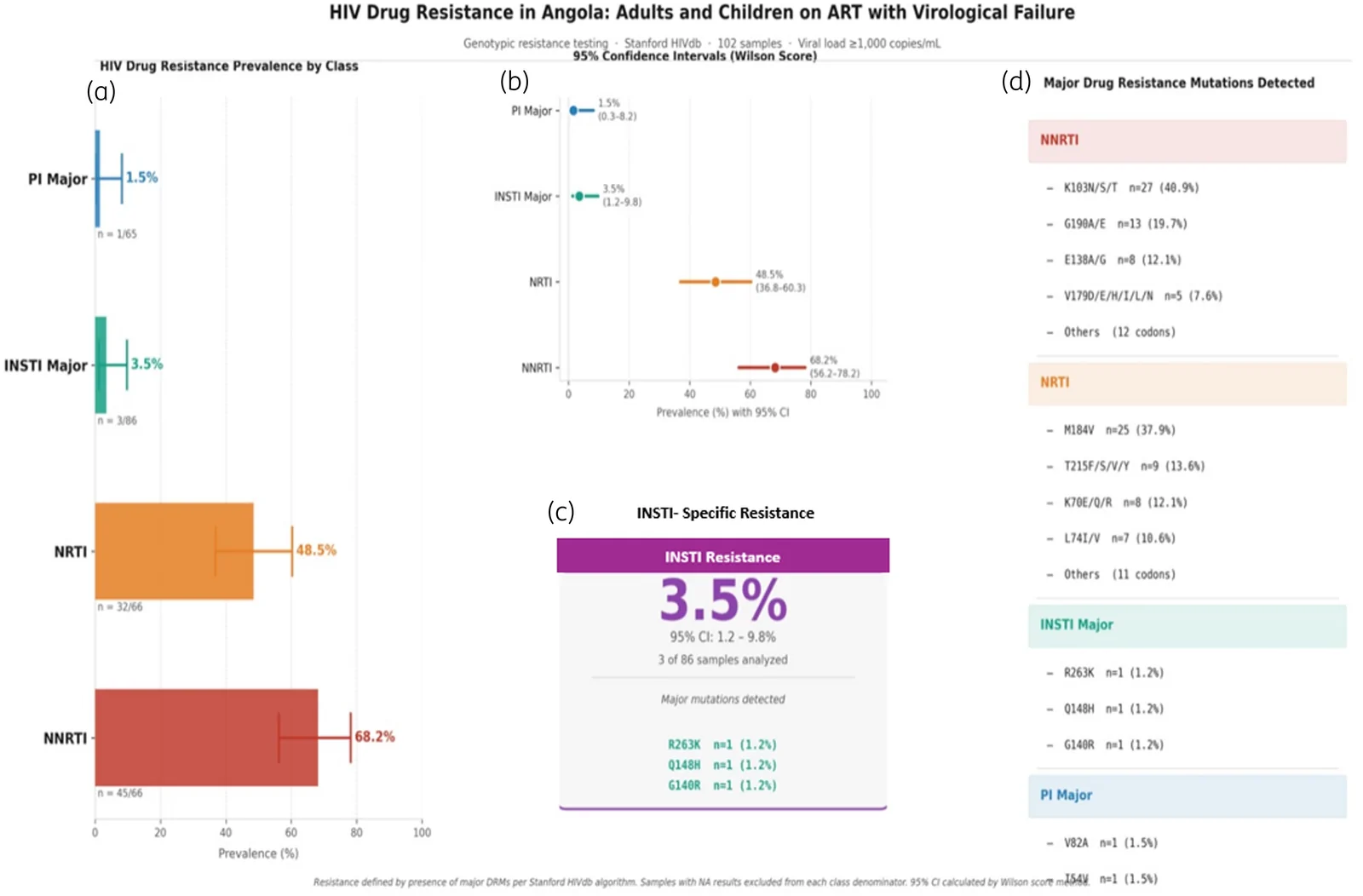
HIV-1 drug resistance in adults and children with viral non-suppression. The figure summarizes: (a) The prevalence of HIV drug resistance according to antiretroviral drug class. (b) The 95% confidence intervals (CIs) for each drug class group. (c) The INSTI resistance prevalence and the corresponding 95% CI. (d) The frequency and prevalence of major DRM detected by class.

Among INP, NNRTI resistance mutations were more frequent than in IEP, particularly K103N/S/T (39.5% versus 18.5%; *P* < 0.0001) and G190A/E (18.7% versus 5.6%; *P* = 0.038), both demonstrating statistically significant differences, whereas E138A/G (10.4% versus 3.7%; *P* = 0.181) did not show statistical significance. A similar pattern was observed for NRTI-associated mutations, which were more common in INP, notably M184V (47.7% versus 5.6%; *P* < 0.0001) and L74V/I (12.5% versus 1.9%; *P* = 0.033), both statistically significant, while K70R/Q/E (12.5% versus 3.7%; *P* = 0.098) was not statistically significant. Regarding INSTI resistance, major mutations G140R and Q148H were identified in INP (both 2.1%), whereas R263K (1.9%) was detected exclusively in IEP. Due to their low frequency, no statistically significant comparisons could be made with these mutations. In contrast, PI-associated resistance mutations were observed only in the IEP group (Figure 2).

**Figure 2.**
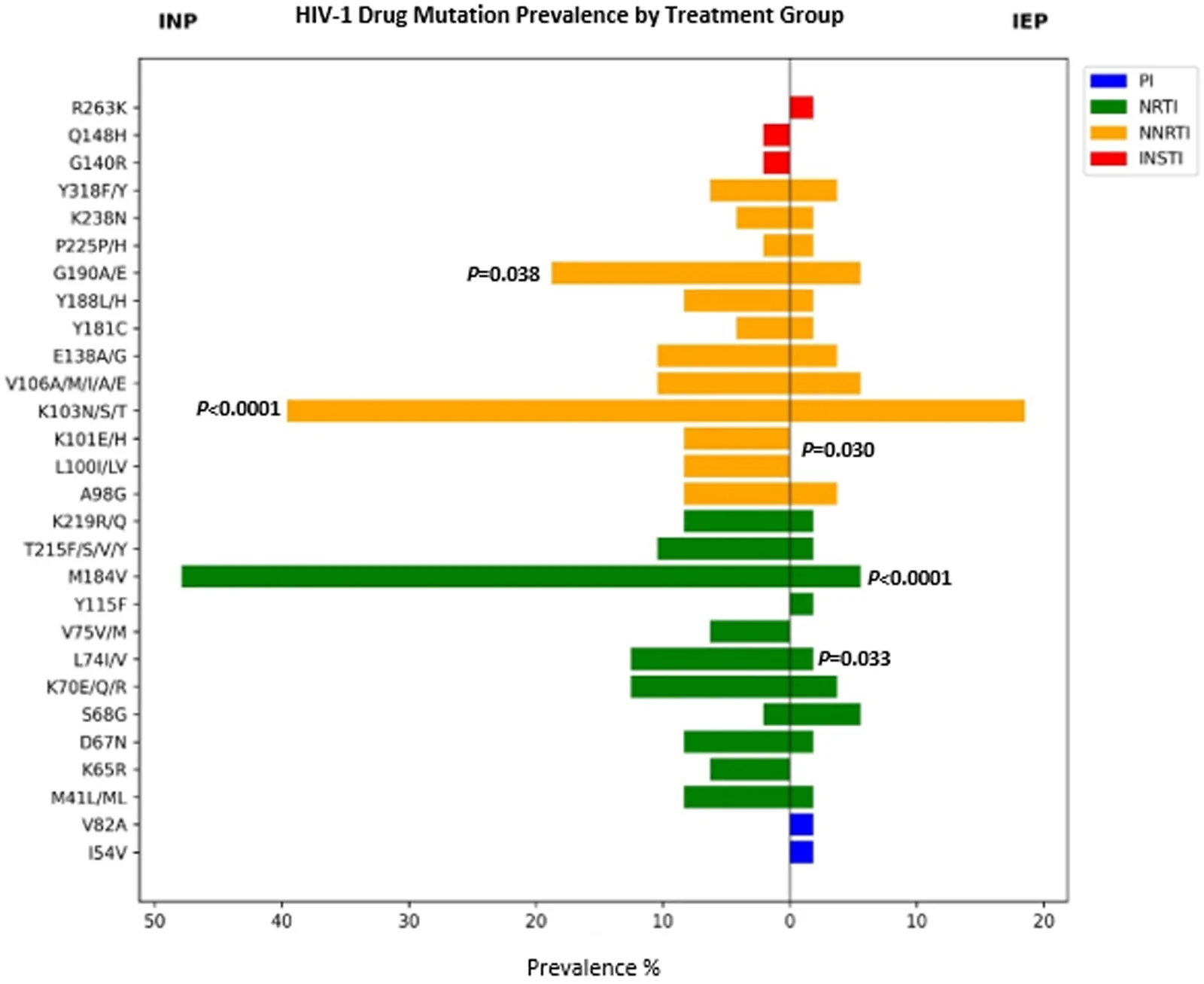
HIV-1 drug mutation prevalence by treatment group. The figure illustrates the comparison of the prevalence of resistance-associated mutation detected in the PR, RT and INT regions among INP and IEP, highlighting the similarities and differences in mutations patterns between the two groups.

For the patients with INSTI DRM, among INP, the samples were from two female adults, aged 33 and 46 years old. Both patients were from provinces located in the northern region of Angola: one from Luanda and the other from Malanje. The 46-year-old patient initiated ART in 2011 and had a documented history of exposure to both NRTI- and NNRTI-based regimens. For the 33-year-old patient, the date of ART initiation and information regarding previous ART regimens were not available. At the time of sample collection, both participants were receiving the tenofovir disoproxil fumarate + lamivudine + efavirenz (TDF + 3TC + EFV) regimen. The IEP was a 19-year-old male adult from Luanda, also in the northern region of Angola. ART was initiated in 2019, and the available treatment history indicated previous exposure to NRTI, NNRTI, and PIs. At the time of specimen collection, he was receiving a DTG-based regimen (TDF + 3TC + DTG). Collectively, the available clinical and demographic data indicated that all tree patients were adults from northern Angola. Overall, among the three patients successful amplification of the INT region was achieved, while amplification of the PR/RT regions was achieved only in the second INP, and the analysis identified the NRTI-associated mutation K70R, as well as the NNRTI-associated mutations A98G, K101E, E138A and G190A. No PI-associated drug resistance was detected in the analysed protease region.

When comparing DRMs between adults and children, a higher prevalence of NNRTI-associated mutations was observed among children. Specifically, mutations E138A (19.0% versus 4.9%; *P* = 0.03), Y188L/H (14.3% versus 2.5%; *P* = 0.02) and Y181C (9.5% versus 1.2%; *P* = 0.04) were significantly more frequent in the paediatric population compared with adults. Similarly, NRTI-associated mutations were more prevalent among children. This difference was particularly notable for M184V (42.8% versus 20.9%; *P* = 0.03). Mutations L74V/I (9.5% versus 4.9%; *P* = 0.42) and Y115F (4.8% versus 0%; *P* = 0.04) were also detected more frequently in children, although the difference for L74V/I did not show statistical significance. In contrast, major INSTI resistance mutations were identified exclusively in adults, including G140R, R263K and Q148H, each occurring at a frequency of 1.2% (*P* = 0.60). Regarding PI-associated resistance mutations, V82A and I54V (4.8%; *P* = 0.04) were observed only in the paediatric group (Figure 3).

**Figure 3.**
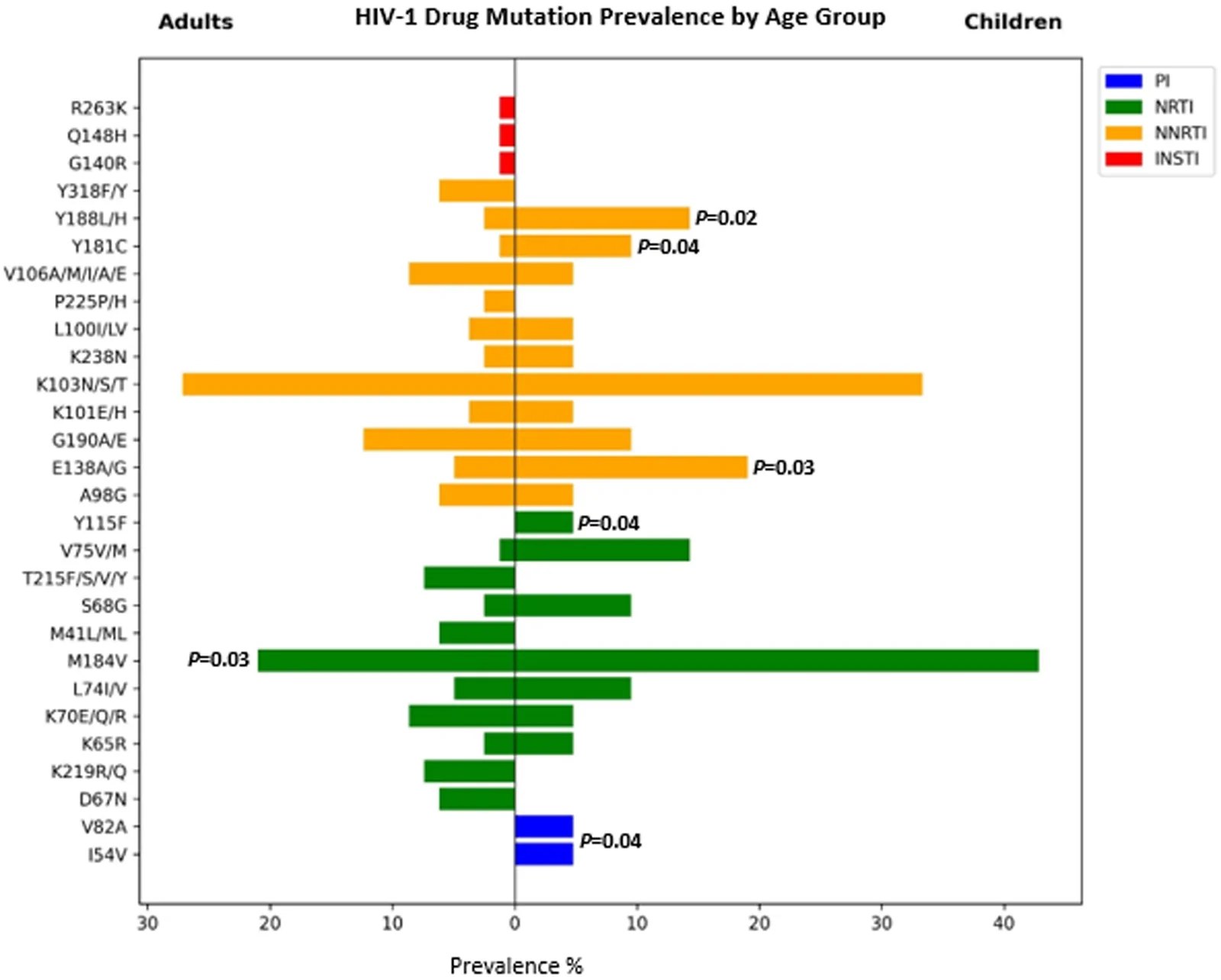
HIV-1 drug mutation prevalence by age group. The figure illustrates the comparison of the prevalence of resistance-associated mutation detected in the PR, RT and INT regions among adult and paediatric patients, highlighting the similarities and differences in mutations patterns between the two age groups.

## Discussion

Understanding the molecular epidemiology of HIV is essential for characterizing viral diversity, monitoring transmission dynamics and informing effective public health interventions. In Angola, however, the current knowledge of HIV genetic diversity and distribution remains limited and geographically uneven. Most previous studies have analysed samples collected in Luanda and a small number of provinces in the country’s central-south region, resulting in a limited representation of the national epidemic.^18–21^ Consequently, the broader molecular landscape of HIV circulating across Angola has not been comprehensively characterized. This lack of geographically representative data constrains our understanding of the distribution and dynamics of circulating viral subtypes and recombinant forms, the prevalence and transmission patterns of DRMs and the epidemiological processes underlying HIV dissemination in the country, limiting the development of evidence-based public health strategies tailored to the Angolan context. In this perspective, the present study aims to contribute to a more representative characterization of HIV genetic diversity, the prevalence of DRMs and the molecular epidemiology of HIV.

In this study, we analysed a cohort of 201 ART-experienced individuals with a median age of 34.0 years, of whom 76.6% were female. This demographic profile is consistent with findings from the Survey of Multiple Health Indicators, which indicate that women aged 15–49 years represent the population most affected by HIV infection in Angola.^22^

The HIV epidemic in the country is characterized by substantial genetic diversity, reflecting a complex molecular epidemiological landscape marked by the co-circulation of multiple subtypes and recombinant forms.^18,19,23^ Our results show that Subtype C was the most prevalent variant (21.6%), followed by URFs (20.6%), A1 (15.7%), F1 (13.7%), CRF02_AG (10.8%), G (8.8%) and others subtypes and recombinant forms with 8.8% of the analysed sequences. This distribution is consistent with previous studies conducted in the country.^21,23^ The predominance of Subtype C reflects the broader epidemiological pattern observed across southern Africa, where this subtype represents the most widely circulating lineage, particularly in countries such as South Africa, Botswana and Zambia.^24,25^ The high proportion of recombinant forms observed in this study likely reflects the long-standing co-circulation of multiple HIV-1 lineages in the region, which creates favourable conditions for viral recombination. Such genetic complexity has been reported previously in central and southern Africa and may contribute to the ongoing emergence of novel recombinant variants.^26^ From a public health perspective, this extensive viral diversity highlights the importance of sustained molecular surveillance to better understand transmission dynamics, monitor the emergence of new recombinant strains and assess their potential implications for treatment response and resistance development.^24^

For many years, first-line ART regimens in Angola relied predominantly on combinations of NRTIs and NNRTIs. Although widely used, NNRTI-based regimens are characterized by a relatively low genetic barrier to resistance, which may facilitate the rapid emergence and accumulation of DRMs, particularly in settings of incomplete viral suppression or suboptimal adherence.^27^ In this context, the high prevalence of DRMs to NNRTIs (68.2%) and NRTIs (48.5%) observed among both adults and children in the present study is consistent with previous reports.^20,21,28^ When comparing DRMs associated with NNRTIs and NRTIs, a higher prevalence is observed among paediatric populations. This pattern can probably be attributable to prior exposure to antiretrovirals administered for the prevention of mother-to-child transmission, which can select resistant viral variants early in life. However, the absence of comprehensive clinical data for the paediatric cohort limits the ability to substantiate this hypothesis and constitutes a key limitation of the study. Additionally, suboptimal adherence, age-specific pharmacokinetic variability and challenges in achieving appropriate dosing in children may further contribute to the emergence and persistence of resistance mutations in this population.^29,30^ The prevalence of DRMs also differed significantly between groups, with INP exhibiting higher frequencies compared to IEP. Evidence indicates that the prevalence of NRTI-associated resistance mutations can decline over time, reflecting both the natural reversion of archived mutations in the absence of selective pressure and the continued ability of NRTIs to suppress viral replication and prevent the emergence of new mutations.^31,32^ Furthermore, the introduction of INSTIs, particularly second-generation ones, has been associated with a marked reduction in the acquisition of additional NRTI and NNRTI mutations.^33–36^

The most frequently observed NNRTI-associated mutations (K103N/S/T, G190A/E and E138A/G) and NRTI-associated mutations (M184V, K70E/Q/R and T215F/S/V/Y) are consistent with the selective pressure exerted by previous antiretroviral regimens used in the country, particularly those including tenofovir, zidovudine, lamivudine, nevirapine and efavirenz. Specifically, K103N/S and G190A are well known to confer high-level resistance to nevirapine and efavirenz, while M184V is associated with high-level resistance to lamivudine and emtricitabine, as well as reduced viral fitness. Additionally, thymidine analogue-associated mutations such as K70R and T215Y are associated with prior exposure to zidovudine and contribute to reduced susceptibility to nucleoside reverse transcriptase inhibitors.^37,38^

Angola initiated the national transition to DTG-based ART in 2021.^13^ This policy shift was implemented to improve treatment durability, enhance tolerability and increase the genetic barrier to resistance compared with previously used regimens. Based on this, the low prevalence of resistance-associated mutations to INSTIs observed in our cohort (3.5%) supports the effectiveness of this strategy and is consistent with the current WHO recommendations that endorse DTG-based regimens as the preferred first-line therapy to mitigate the growing burden of HIVDR in many LMICs.^18^ Nevertheless, given the relatively recent introduction of DTG-based regimens in the national programme, continued molecular surveillance will be essential to monitor the potential emergence and transmission of INSTI resistance over time.^39,40^

In this study, despite being unexpected, two major resistance-associated mutations to INSTIs, G140R and Q148H, were identified in two distinct patients from the INP cohort, whereas a major mutation, R263K, was detected in a patient from the IEP cohort. Consistent with the broader literature, the prevalence of INSTI-associated DRMs remains low, even in regions where INSTIs have been widely implemented in ART for several years. When such mutations are detected in INSTI-naive individuals, they likely represent a combination of true transmitted INSTI resistance and the low baseline frequency of rare, naturally occurring non-polymorphic mutations.^41^ Clinical evidence indicates that mutations at position Q148, particularly when present in combination with other INSTI resistance mutations, are associated with reduced virological suppression in individuals receiving DTG-based ART.^42,43^ Case reports have also documented the emergence of Q148H and V151V/L mutations in ART-experienced but INSTI-naive patients who failed first-line NNRTI-based regimens, highlighting the potential for prior ART exposure to influence the resistance profile even before INSTI exposure.^44^

Protease inhibitors remain a critical therapeutic option for patients experiencing virological failure on DTG-based regimens. In the present study, no major darunavir resistance-associated mutations were detected, while PI mutations I54V and V82A conferring resistance to lopinavir and atazanavir were identified in only 1.5% of the analysed sequences. These findings are consistent with global evidence indicating that PI resistance remains relatively uncommon, largely due to the high genetic barrier to resistance and the limited cross-resistance profiles characteristic of this drug class.^43^ Maintaining sustained access to boosted PI regimens, together with their rational and guideline-directed use, will therefore be essential to preserve the effectiveness of second-line therapeutic strategies. In addition, strengthening routine resistance surveillance and ensuring timely optimization of treatment for individuals experiencing virological failure will be critical to sustaining long-term treatment success and limiting the accumulation of drug resistance.^8,43^

### Limitations

This study has certain limitations that should be acknowledged. The lower amplification rate of the PR/RT and INT coding regions observed mostly in DBS samples can likely be attributed to their prolonged storage and exposure to multiple freeze-thaw cycles, factors known to compromise nucleic acid integrity and reduce amplification efficiency. In addition, the possibility that some specimens were stored under suboptimal conditions may have further contributed to decreased sequencing performance resulting in a non-uniform sequencing success across the same patients for the PR, RT and INT regions and the results cannot be directly compared or combined to infer cross-resistance patterns. However, the overall prevalence of these mutations was low, suggesting a minimal impact on the interpretation of resistance patterns and the study’s conclusions. Importantly, the internal consistency of the data and their concordance with established scientific evidence support the robustness, reliability and scientific validity of the findings despite these constraints.

### Conclusions

The HIV epidemic in Angola is characterized by considerable epidemiological and genetic complexity, reflected in the co-circulation of multiple subtypes and recombinant forms. In the context of the rapid scale-up of DTG-based ART in many LMICs, including the national treatment programmes, the continued monitoring of INSTI resistance mutations is increasingly important. Given the current limitations in genotypic resistance surveillance capacity, strengthening molecular monitoring systems will be critical to detect the potential emergence and transmission of INSTI resistance. Such surveillance is essential not only to sustain long-term treatment efficacy but also to provide evidence-based public health strategies aimed at preventing the spread of transmitted drug resistance and optimizing ART regimens at the population level.
